# Serpin Family B Member 2 Polymorphisms in Patients with Diabetic Kidney Disease: An Association Study

**DOI:** 10.3390/ijms252010906

**Published:** 2024-10-10

**Authors:** Maria Tziastoudi, Georgios Pissas, Spyridon Golfinopoulos, Georgios Filippidis, Christina Poulianiti, Evangelia E. Tsironi, Efthimios Dardiotis, Theodoros Eleftheriadis, Ioannis Stefanidis

**Affiliations:** 1 Department of Nephrology, School of Medicine, University of Thessaly, Panepistimiou 3, Biopolis, 41500 Larissa, Greece; matziast@med.uth.gr (M.T.); gpissas@msn.com (G.P.); spygolfin@yahoo.gr (S.G.); gfilippid@yahoo.gr (G.F.); christinepoulianiti@yahoo.gr (C.P.); teleftheriadis@med.uth.gr (T.E.); 2 Ophthalmology Clinic, University of Thessaly, 41335 Larissa, Greece; e_tsironi@hotmail.com; 3 Neurology, General University Hospital of Larissa, 41500 Larissa, Greece; edar@med.uth.gr

**Keywords:** diabetes mellitus, diabetic kidney disease, Serpin Family B Member 2 (*SERPINB2*), polymorphism, association study

## Abstract

Diabetic kidney disease (DKD) is a serious microvascular complication of type 2 diabetes mellitus (T2DM). Despite the numerous genetic loci that have been associated with the disease in T2DM, the genetic architecture of DKD remains unclear until today. In contrast to *SERPINE1*, the contribution of *SERPINB2* has not been examined in DKD. Therefore, we conducted the first genetic association study of *SERPINB2* to elucidate its role in DKD. In total, the study involved 197 patients with DKD, 155 patients with T2DM without microvascular complications (diabetic kidney disease, diabetic retinopathy, and diabetic neuropathy), and 246 healthy controls. The generalized odds ratio (OR_G_) was calculated to estimate the risk on DKD development. The present association study regarding *SERPINB2* SNPs (rs4941230, rs3819335, rs13381217, rs6140) did not reveal any significant association between *SERPINB2* variants and DKD. Additional studies in other populations are necessary to further investigate the role of this gene in the progression of diabetes mellitus and development of DKD.

## 1. Introduction

Diabetic kidney disease (DKD), often referred to as diabetic nephropathy, represents a significant microvascular complication of diabetes, standing as the foremost contributor to end-stage kidney disease on a global scale [1,2]. Type 2 diabetic kidney disease (T2DKD) is a prevalent microvascular complication of type 2 diabetes mellitus (T2DM), with its occurrence steadily on the rise alongside the continual increase in T2DM prevalence [3].

DKD is influenced by many factors. Current research in the field of T2DKD suggests its connection with disruptions in glucose and lipid metabolism [4], alterations in kidney hemodynamics [5], aberrant activation of the renin–angiotensin–aldosterone system [6], inflammatory responses [7], oxidative stress [8], genetic predisposition factors [9,10], as well as epigenetic mechanisms [11].

To shed light on the genetic dissection of DKD, researchers have carried out numerous studies with various methodological designs. Before the era of genetic association studies, genetic linkage studies were conducted which were either hypothesis driven or genome-wide [12,13,14,15,16,17,18]. Similar to linkage studies, before large-scale GWAS became affordable and feasible, numerous candidate genetic association studies were conducted for DKD [19,20,21,22,23]. However, these studies predominantly yielded limited and inconsistent findings.

Serine proteinase inhibitors (serpins) are intracellular proteins, while most of their identified targets are extracellular. They are distinguished by their unique mechanism of action, in which they irreversibly inhibit their target protease by undergoing a significant conformational change that disrupts the target’s active site. Serpins regulate a variety of biological processes, such as coagulation and inflammation, through protease inhibition [24].

Serine Proteinase Inhibitor 2 (*SERPINB2*), also named Plasminogen Activator Inhibitor 2 (PAI-2), is a member of the clade B of the serpin superfamily [25,26]. It constitutes a coagulation factor that inactivates tissue plasminogen activator and urokinase and is expressed in various normal and transformed cell types, especially following stimulation by inflammatory cytokines [27]. Immune cells possessing serpins include granulocytes, monocytes, and cytotoxic lymphocytes [28]. SerpinB2 is increasingly recognized as a novel regulator of macrophage survival, with its deficiency linked to impaired CCL2-mediated macrophage influx into the small intestine [29]. A growing number of factors, including immune signals (such as LPS and Th2 cytokines) and hormonal signals (like gastrin and 5-HT), are associated with increased serpinB2 expression [30]. Consequently, serpinB2 is an immune-regulated factor with multiple roles in the intestinal mucosa. There is also evidence that serpinB2 is involved in adaptive immune responses. The production of serpinB2 in macrophages significantly increases during microbial, viral, and nematode infections [31,32,33].

A few studies have investigated the role of PAI-1 in the risk of DKD, but the results remain conflicting. However, no study has investigated the role of *PAI*-*2* in the pathogenesis of DKD. In this study, to elucidate the contribution of the *SERPINB2* gene to the pathogenesis of diabetic kidney disease in the context of type 2 diabetes mellitus (T2DM), we selected four tag single-nucleotide polymorphisms (SNPs) for genotyping in a case-control study of Caucasians.

## 2. Materials and Methods

### 2.1. Study Population

The study protocol was approved by the Ethics Committee of the University Hospital of Larissa, University of Thessaly, School of Medicine. Conducted at the University Hospital of Larissa, all participants provided informed consent before enrolment. Participants were recruited between 2009 and 2011 from the outpatient wards of Nephrology, Internal Medicine, and Ophthalmology at the University Hospital of Larissa. They were all Caucasians of Greek origin and resided in the same region in central Greece (Thessaly) during the study.

The study design and participant details have been described elsewhere [21]. In summary, the study involved patients with longstanding (>10 years) type 2 diabetes mellitus (T2DM), more specifically, 197 patients with diabetic nephropathy (DN) and 155 patients with type 2 diabetes mellitus (T2DM) without microvascular complications (diabetic kidney disease, diabetic retinopathy, and diabetic neuropathy), as well as 246 healthy controls. All participants were examined at the Ophthalmology and Nephrology outpatient clinics of the University Hospital of Larissa in Greece.

Diabetic kidney disease (DKD) patients were selected by laboratory examination and biopsy was not performed. DKD was characterized by persistent albuminuria, defined as urinary albumin excretion over 300 mg/24 h (>200 μg/min), independent of serum creatinine levels, determined on at least two separate occasions three months apart from one another and in the absence of clinical or radiological evidence of non-diabetic renal disease.

### 2.2. Genotyping

Genomic DNA was extracted from peripheral blood samples using a salting-out method. Based on the HapMap population database for Utah residents with Northern and Western European ancestry (CEU) (Release 27, Phase II + III, Feb09, on NCBI B36 assembly, dbSNP b126), tag single nucleotide polymorphisms (SNPs) across *SERPINB2* were identified based on linkage disequilibrium (LD) blocks according to the HapMap project (http://hapmap.ncbi.nlm.nih.gov, accessed on 15 January 2024) using the Tagger genetic program (http://www.broadinstitute.org/mpg/tagger, accessed on 15 January 2024). Tagging SNPs were selected using criteria of an r^2^ cut-off of ≥0.8 and a minor allele frequency (MAF) of >0.05. A total of 4 SNPs (rs4941230, rs3819335, rs13381217, rs6140) were retrieved.

Genotyping of the tag SNPs was conducted using TaqMan allele-specific discrimination assays on an ABI PRISM 7900 sequence detection system, analyzed with SDS software version 2.3 (Applied Biosystems, Foster City, CA, USA). The laboratory personnel were blinded to the clinical status of the participants.

### 2.3. Data Analysis

Continuous variables were expressed as mean ± standard deviation (SD), while nominal variables were presented as count and percentage [n (%)]. The normality of continuous variables was assessed using the Kolmogorov–Smirnov test. Pairwise comparisons of continuous variables were conducted using either the t-test or the Mann–Whitney U test for unpaired data, as appropriate. Frequencies of categorical variables were compared using the χ^2^ test or Fisher’s exact test.

The association between genotype distribution and disease was analyzed using the generalized odds ratio (OR_G_) [34,35]. The association between genotype distribution and the disease status (i.e., healthy controls, diseased controls and cases) was additionally tested using the χ^2^ test. In healthy controls, the genotype distribution was tested for deviation from Hardy–Weinberg equilibrium (HWE).

Genotype distribution and statistical analysis were calculated using IBM^®^ SPSS^®^ Statistics Version 29 (IBM Corp.© (Armonk, NY, USA), Release 29.0.2.0, 2024). The OR_G_ was calculated using ORGGASMA (http://biomath.med.uth.gr, accessed on 30 June 2024).

## 3. Results

### 3.1. Clinical Profile of Participants

The cohort included 197 cases (patients with T2DM and DN), 155 controls with disease (patients with T2DM without DN), and 246 healthy controls, all of whom were Caucasians of Greek origin. The demographic and clinical characteristics are presented in Table 1, as has been described elsewhere [22]. All patients were receiving treatment for chronic kidney disease, diabetes, and hypertension, including angiotensin-converting enzyme (ACE) inhibitors and angiotensin receptor blockers (ARBs) as needed.

### 3.2. Genotype Distribution and Severity of T2DM

In patients with diabetic nephropathy (DN; cases) in contrast to patients with T2DM without microangiopathy (microvascular complications; controls with disease) there is an enhanced clinical severity of T2DM. The genotype distributions of the four variants according to severity of T2DM in cases, controls with disease, and healthy controls are shown in Table 2, whereas the respective OR_G_ are shown in Table 3. The healthy controls were conformed to HWE for all variants (*p* ≥ 0.05), except rs13381217. There was not a significant association between disease progression and genotype distribution of certain *SERPINB2* variants. The model-free approach (OR_G_) did not produce significant results for these variants, indicating that the risk of disease severity is not related to the mutational load of these variants (i.e., subjects with disease have higher mutational load than healthy controls). We also examined the association between the four variants and disease severity considering all possible comparisons (Table 3).

## 4. Discussion

This study examined whether specific variants in the *SERPINB2* gene, encompassing four tag SNPs, are linked to the progression of type 2 diabetes mellitus and the development of diabetic kidney disease in the context of this type of diabetes mellitus. Our analysis did not reveal any significant association between *SERPINB2* variants and DKD, indicating no implication of *SERPINB2* variants in the risk or development of the disease.

*SERPINB2* has been investigated in numerous diseases. For instance, the inhibition of this serine protease by physiological inhibitors is expected to reduce invasion and metastasis [36]. There is a significant association between SerpinB2 levels and survival, with breast cancer cell-associated SerpinB2 being identified as an unfavorable prognostic indicator [37,38,39], although the exact mechanism remains unclear.

It has also been observed that the expression of *SERPINB2* is significantly increased under various inflammatory conditions. More specifically, *SERPINB2* has been identified as the most upregulated gene in peripheral blood monocytes of patients with inflammatory bowel disease [40,41] and in monocytes of patients with systemic lupus erythematosus [42]. Additionally, it has been reported that *SERPINB2* plays a role in regulating inflammatory responses in psoriasis [43]. However, the precise molecular mechanisms linking *SERPINB2* to the immune response still need to be identified.

Our study design has several strengths, one of which is a clear case definition. Patients without persistent proteinuria were not classified as having DN. Additionally, we included healthy controls without diabetes to identify variants associated with diabetes mellitus but not specifically with DN. Furthermore, the use of OR_G_ is a model-free approach, leveraging the full genotype distribution and offering a straightforward interpretation of genetic association.

However, certain limitations of this study must be acknowledged. First, the relatively small sample size may lead to false positive and false negative results, which is a common limitation in many candidate GASs. Second, adjusting for other potential confounders would provide more robust and accurate results. In addition, non-proteinuric diabetic kidney disease (NPDKD) was not included in the cases. This fact could be a problem of stratification as proteinuria alone may not be sufficient to capture the full spectrum of kidney disease in diabetes. Alternative pathways, like tubulointerstitial damage or ischemia, which do not primarily involve proteinuria, may be involved in diabetic nephropathy. Therefore, additional proximal tubular biomarkers (e.g., NGAL, KIM-1) or imaging techniques might provide better tools for stratifying patients more accurately and might be helpful in defining normoalbuminuric DKD. Stratification of patients was based on proteinuria and albuminuria in 24 h urine. Instead, urinary protein-to-creatinine ratio (PCR) or albumin-to-creatinine ratio (ACR) could be implemented with some diagnosis and stratification advantage [44]. Histology would be the gold standard for patient stratification in this study; however, kidney biopsy is not standard care in patients with T2DM.

The lack of a significant association between *SERPINB2* variants and DKD may indicate that these genetic variants do not have a major role in the progression of T2DM and in the risk or development of DKD. This could be since *SERPINB2* is involved in fibrinolysis, a pathway that may not be central to the processes that cause diabetic kidney damage. More specifically, in DKD, the primary pathological processes include glomerular damage (e.g., podocyte injury, basement membrane thickening) and tubulointerstitial damage (e.g., fibrosis, tubular atrophy). *SERPINB2*′s role in regulating inflammation and coagulation may not directly influence these mechanisms, which might explain the lack of a significant association. Other explanations could include insufficient statistical power, the heterogeneity of T2DM and DKD, the fact that the majority of the variants studied having no functional consequences, or the fact that DKD is driven by other genetic pathways, such as those related to inflammation, oxidative stress, and metabolic dysfunction. In addition, *SERPINB2* variants may not have a large enough effect on their own to contribute significantly to DKD risk, especially in the presence of stronger genetic and environmental factors. For instance, variants in genes related to inflammatory cytokines, oxidative stress, and metabolic regulation might be more strongly linked to DKD risk than those related to fibrinolysis regulation.

Therefore, further studies involving larger samples and diverse ethnic groups are necessary to clarify the role of the *PAI-2* gene in T2DM progression and DKD development.

## Figures and Tables

**Table 1:** Clinical profile of the participants.

Parameters	Case-Control Study Population Groups (*n* = 498)					
	HC	DM	*p* Value *	DM-DN	DM + DN	*p* Value *
N	246	352	n.a.	155	197	n.a.
Sex [m; n (%)]	136 (55.3)	181 (51.4)	0.361	74 (47.7)	107 (54.3)	0.238
Age (years)	71 ± 9.2	68 ± 8.9	<0.001	68 ± 9.1	69 ± 8.8	0.427
DM duration (years)	n.a.	16.3 ± 8.0	n.a.	15.7 ± 8.3	16.8 ± 7.8	0.508
HbA1c (%)	n.a.	7.35 ± 1.31	n.a.	7.20 ± 1.34	7.47 ± 1.29	0.019
Insulin treatment (%)	n.a.	105 (29.8)	n.a.	50 (32.3)	55 (27.9)	0.412
Hypertension (%)	0	224 (63.6)	<0.001	98 (63.2)	126 (63.9)	0.911
Cardiovascular disease (%)	0	110 (31.3)	<0.001	41 (26.5)	69 (35.0)	0.105
Creatinine (mg/dl)	0.77 ± 0.15	1.46 ± 1.37	<0.001	0.90 ± 0.18	1.84 ± 1.67	<0.001
Urea (mg/dl)	30 ± 7.9	59 ± 34	<0.001	42 ± 13.6	71 ± 38.3	<0.001
Albuminuria (mg/d)	n.a.	470 ± 856	n.a.	43.9 ± 53.3	782 ± 1019	<0.001
Proteinuria (mg/d)	n.a.	788 ± 1468	n.a.	105 ± 80.0	788 ± 1468	<0.001

Continuous data are given as mean and standard deviation [x  ±  SD] and categorical data as count and percentage [*n* (%)]. * *p*-values were calculated by the Mann–Whitney *U* test for continuous variables or the *χ*^2^ test for categorical variables as appropriate. HC: healthy controls, DM: diabetics, DM + DN: diabetics with diabetic nephropathy, DM-DN: diabetics without diabetic nephropathy. n.a.: not applicable.

**Table 2:** Genotype frequencies of the participants.

Variant	Genotype	DM + DN (n)	DM-DN (n)	HC (n)
rs4941230	A A	119	93	146
	A G	61	49	87
	G G	14	7	11
rs3819335	T T	112	92	139
	T A	66	55	79
	A A	9	7	17
rs13381217	C C	187	137	226
	C T	6	9	10
	T T	0	0	2
rs6140	C C	115	90	137
	C G	68	55	80
	G G	9	6	18

DM + DN: diabetics with diabetic nephropathy, DM-DN: diabetics without diabetic nephropathy, HC: healthy controls.

**Table 3:** Results of the association study.

	DN versus DM	DN versus HC	DN versus DM versus HC	DM versus HC
SNP	OR (95% CI)	OR (95% CI)	OR (95% CI)	OR (95% CI)
rs4941230	1.08 (0.72, 1.63)	0.99 (0.69, 1.42)	0.98 (0.76, 1.28)	0.91 (0.61, 1.36)
rs3819335	1.00 (0.66, 1.51)	0.94 (0.65, 1.35)	0.95 (0.74, 1.24)	0.94 (0.64, 1.38)
rs13381217	0.50 (0.18, 1.39)	0.65 (0.25, 1.72)	0.79 (0.44, 1.43)	1.29 (0.54, 3.09)
rs6140	1.00 (0.66, 1.51)	0.90 (0.63, 1.30)	0.93 (0.71, 1.20)	0.90 (0.61, 1.33)

DN: diabetics with diabetic nephropathy, DM: diabetics without diabetic nephropathy, HC: healthy controls.

## Data Availability

The datasets used and/or analysed during the current study are available from the corresponding author on reasonable request.
